# Population trends of seabirds in Mexican Islands at the California Current System

**DOI:** 10.1371/journal.pone.0258632

**Published:** 2022-10-07

**Authors:** Federico Méndez Sánchez, Yuliana Bedolla Guzmán, Evaristo Rojas Mayoral, Alfonso Aguirre-Muñoz, Patricia Koleff, Alejandro Aguilar Vargas, Fernando Álvarez Santana, Gustavo Arnaud, Alicia Aztorga Ornelas, Luis Felipe Beltrán Morales, Maritza Bello Yáñez, Humberto Berlanga García, Esmeralda Bravo Hernández, Ana Cárdenas Tapia, Aradit Castellanos Vera, Miguel Corrales Sauceda, Ariana Duarte Canizales, Alejandra Fabila Blanco, María Félix Lizárraga, Anely Fernández Robledo, Julio César Hernández Montoya, Alfonso Hernández Ríos, Eduardo Iñigo-Elias, Ángel Méndez Rosas, Braulio Rojas Mayoral, Fernando Solís Carlos, Alfredo Ortega-Rubio

**Affiliations:** 1 Grupo de Ecología y Conservación de Islas, A. C., Ensenada, Baja California, México; 2 Centro de Investigaciones Biológicas del Noroeste, S. C., La Paz, Baja California Sur, México; 3 Comisión Nacional para el Conocimiento y Uso de la Biodiversidad, Ciudad de México, México; 4 BirdLife International, Americas Partnership Secretariat, Quito, Ecuador; 5 Cornell Laboratory of Ornithology, Ithaca, New York, United States of America; MARE – Marine and Environmental Sciences Centre, PORTUGAL

## Abstract

The Baja California Pacific Islands (BCPI) is a seabird hotspot in the southern California Current System supporting 129 seabird breeding populations of 23 species and over one million birds annually. These islands had a history of environmental degradation because of invasive alien species, human disturbance, and contaminants that caused the extirpation of 27 seabird populations. Most of the invasive mammals have been eradicated and colonies have been restored with social attraction techniques. We have recorded the number of breeding pairs annually for most of the colonies since 2008. To assess population trends, we analyzed these data and show results for 19 seabird species on ten island groups. The maximum number of breeding pairs for each nesting season was used to estimate the population growth rate (λ) for each species at every island colony. We performed a moving block bootstrap analysis to assess whether seabird breeding populations are increasing or decreasing. San Benito, Natividad, and San Jerónimo are the top three islands in terms of abundance of breeding pairs. The most widespread species is Cassin’s Auklet (*Ptychoramphus aleuticus*) with 14 colonies. Thirty-one populations of 14 species are significantly increasing while eleven populations of seven species are decreasing. We did not find statistical significance for 19 populations, however, 15 have λ>1 which suggest they are growing. Twelve of the 18 species for which we estimated a regional population trend are significantly increasing, including seven surface-nesting species: Brandt’s Cormorant (*Phalacrocorax penicillatus*), Brown Pelican (*Pelecanus occidentalis*), Caspian Tern (*Hydroprogne caspia*), Double-crested Cormorant (*P*. *auritus*), Elegant Tern (*Thalasseus elegans*), Laysan Albatross (*Phoebastria immutabilis*) and Western Gull (*Larus occidentalis*), and five burrow-nesting species: Ainley’s (*Hydrobates cheimomnestes*), Ashy (*H*. *homochroa*) and Townsend’s (*H*. *socorroensis*) Storm-Petrels, and Craveri’s (*Synthliboramphus craveri*) and Guadalupe (*S*. *hypoleucus*) Murrelets. The BCPI support between 400,000 and 1.4 million breeding individuals annually. Our results suggest that these islands support healthy and growing populations of seabirds that have shown to be resilient to extreme environmental conditions such as the “Blob”, and that such resilience has been strengthen from conservation and restoration actions such as the eradication of invasive mammals, social attraction techniques and island biosecurity.

## Introduction

Of all the birds in the world, seabirds are the most threatened group and the one with the greatest and fastest declines [1–4]. Invasive alien species are the prevalent threat to seabirds, followed by fisheries bycatch, climate change and severe weather, pollution, and human disturbance [2,5]. North American seas and islands support nearly half of all seabird species globally [2,6], mostly due to the high productivity associated with the California Current System (CCS) [7–10]. This highly productive upwelling ecosystem is a hotspot for seabirds, where their abundance and population trends have been long and well-studied in the Canadian and United States portions of the CCS [9–12] but not further south in Mexico in a comprehensive and systematic manner. For the Mexican portion of the CCS there is no exhaustive assessment of at-sea seabird abundances while the last regional multispecies population estimates for colonies in the Baja California Pacific Islands (BCPI; from Coronado Archipelago in the north to the Magdalena Bay islands in the south) are from the period 1999–2003, when the BCPI harbored half (2,433,000) of the breeding individuals and 22 out of 37 taxa that occurred in the whole CCS [13].

The BCPI are home to a fifth of the world’s 368 seabird species [2,5,14]. Fourteen (18%) of the 80 seabird species in the BCPI are threatened: two species as Critically Endangered, three as Endangered, and nine as Vulnerable on the IUCN Red List of Threatened Species (IUCN 2021), while 23 (29%) are federally listed in Mexico’s Official Norm for species at-risk (*NOM-059-SEMARNAT-2010*) [14,15]. In terms of endemism (i.e., endemic and semi-endemic species) [16], the BCPI host colonies of 10 of the 12 species endemic to Mexico: Craveri’s Murrelet (*Synthliboramphus craveri*), Guadalupe Murrelet (*S*. *hypoleucus*), Ainley’s Storm-Petrel (*Hydrobates cheimomnestes*), Black Storm-Petrel (*H*. *melania*), Guadalupe Storm-Petrel (*H*. *macrodactylus*), Least Storm-Petrel (*H*. *microsoma*), Townsend’s Storm-Petrel (*H*. *socorroensis*), Elegant Tern (*Thalasseus elegans*), Heermann’s Gull (*Larus heermanni*), and Black-vented Shearwater (*Puffinus opisthomelas*) [15,17].

Most of the seabird populations on the BCPI were severely reduced during the 20th century [13,18], with 27 seabird populations extirpated due to the combined negative effects of invasive alien mammals and direct human disturbance [13,17,19]. Much has changed for seabird conservation in Mexico during the last couple of decades, particularly in the southernmost region of the CCS (i.e., the Pacific Ocean off the Baja California Peninsula). From an almost complete lack of knowledge and inaction by the late 90’s, where little was known about seabird populations [19,20] and no protection of their colonies existed [13,21], Mexico has taken bold conservation actions, including the legal protection of its nearly 4,500 islands [22], the removal of invasive mammals from 39 islands [18,23], the restoration and long-term monitoring of seabird populations [17,24,25], and the formulation of a National Action Program for Seabird Conservation (*PACE Aves Marinas*) [15].

Benefits derived from the eradication of invasive mammals on the biodiversity of the world’s islands have been greatly documented [26–29], and many studies have focused on the recovery of seabird populations [30–32]. Mexico is among the countries that have successfully carried out the most eradications of invasive mammals globally with 60 populations from 39 islands [18,23,27,33]. It is also some of the few that actively conduct pre- and post-eradication monitoring [34,35] to assess ecological outcomes [17,18,24,36] as well as active restoration such as seabird social attraction [37–39] once invasive mammals are removed to maximize conservation gains. Benefits to seabird populations in Mexico from both passive and active restoration have also been documented, with the encouraging outcome that to date 23 out of 27 (85%) historically extirpated seabird colonies have been restored, and 12 new colonies have been recorded [17].

Beyond updating the number of breeding individuals at their island colonies [13,17,24,40–43], there have not been any attempts to understand the dynamics of the BCPI seabird populations and to assess recovery or decline trends at the subpopulation and metapopulation level [44,45]. Therefore, building upon our long-term monitoring of the seabird populations on the BCPI for nearly two decades, **in this paper we aim to answer two questions: (1) What is the current abundance of the seabird populations on the BCPI? and (2) What are their population trends?** By doing so, we expect to highlight the importance and the contributions of these populations and their habitats—islands and the surrounding marine environment within the CCS—in the context of seabird conservation in the central eastern Pacific and the world.

## Methods

### Study area

**Fig 1:**
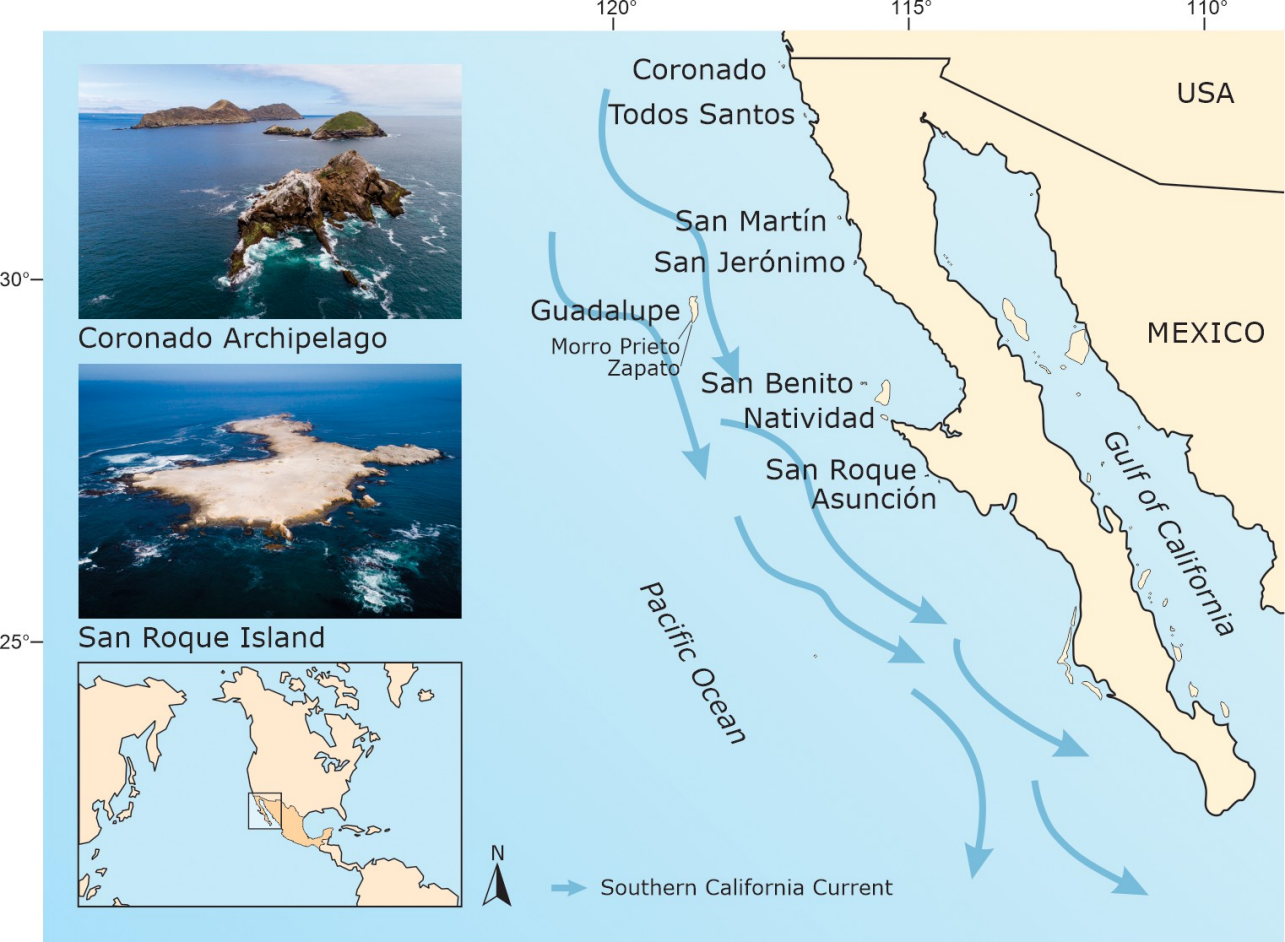
Map of the Baja California Pacific Islands, a seabird hotspot where breeding populations have been systematically monitored for almost two decades. Photos of Coronado Archipelago and San Roque islands are shown—being the extremes in geographic location—to show the heterogeneous physiography of the region’s islands. Arrows depict the southerly flow of the California Current [51], which has significant influence on the region’s marine productivity and thus the seabird populations. Map design credits: © GECI / Gabriela Fernández Ham. Photo credits: © GECI / J.A. Soriano.

The Baja California Pacific Islands (BCPI) are in the southern California Current System, off the Baja California Peninsula (Fig 1). In this region, there are around 30 islands, all within Protected Areas managed by Mexico’s Federal Government through the National Commission for Natural Protected Areas (CONANP): El Vizcaíno Biosphere Reserve; Guadalupe Island Biosphere Reserve; and Baja California Pacific Islands Biosphere Reserve [22,46]. They are key reproduction sites for 133 species of vertebrates: 41 amphibians and reptiles, 69 birds, 19 mammals and four pinnipeds [47]. We focus our analyses on the ten islands and archipelagos described in Table 1. Due to their relevance for birds, all these islands are Important Bird and Biodiversity Areas (IBAs) [48]. The size of the islands where we conducted our work ranges from 35 to 24,171 hectares, with maximal altitudes ranging from 10 to 1,298 meters. Except for San Martín and Guadalupe islands, which have a volcanic origin, the rest are an unsubmerged extension of the continental shelf [49]. Most of them are within proximity to the Baja California Peninsula, between 1.8 to 72 km, with Guadalupe Island and Morro Prieto and Zapato islets being the most oceanic at 260 km. This region is characterized by a Mediterranean climate, with hot and dry summers and cold and wet winters, a regional average annual temperature of 18-23°C, and an average annual cumulative precipitation of *ca*. 200 millimeters. The dominant plant communities are maritime desert scrub, although Guadalupe Island sustains a temperate forest because of its high elevation and an almost permanent fog system [50].

**Table 1:** Characteristics of the Baja California Pacific Islands where breeding seabird populations were monitored during the period 2003–2019.

Island/Archipelago	Area (Hectares)	Protected Area^a^	No. breeding seabird species^b^
Coronado (3 islands, 1 islet)	173	PIBR	11
Todos Santos (2 islands)	123	PIBR	8
San Martín	265	PIBR	10
San Jerónimo	48	PIBR	11
Guadalupe	24,171	GIBR	5
Morro Prieto and Zapato (2 islets)	45	GIBR	8^c^
San Benito (3 islands)	610	PIBR	13
Natividad	736	EVBR	7
San Roque	35	EVBR	9
Asunción	43	EVBR	7

^**a**^PIBR: Baja California Pacific Islands Biosphere Reserve; GIBR: Guadalupe Island Biosphere Reserve; EVBR: El Vizcaíno Biosphere Reserve.

^**b**^Updated from: [13,17,41–43] and present work.

^c^Morro Prieto and Zapato host 6 and 7 species each; collectively, they harbor 8 species (5 shared between them).

To conduct our fieldwork, we obtained permits from all relevant Mexican authorities: (1) Ministry of Interior *(Secretaría de Gobernación–SEGOB*) to visit the islands; (2) General Directorate of Wildlife from the Ministry of Environment and Natural Resources (*Dirección General de Vida Silvestre–DGVS*, *Secretaría de Medio Ambiente y Recursos Naturales–SEMARNAT*) for scientific collection, including the handling of seabirds and biological samples collection; and (3) National Commission for Natural Protected Areas (*Comisión Nacional de Áreas Naturales Protegidas–CONANP*) to conduct fieldwork on islands within natural protected areas. In some cases, particularly for Guadalupe Island, we had the logistical support of the Mexican Navy (*Secretaría de Marina–SEMAR*) for transport of personnel and equipment.

### Monitoring of seabird colonies

We have been monitoring seabird populations on Guadalupe Island since 2003 [24,52], on Asunción and San Roque islands since 2008 [17,23], and from 2014 expanded to the rest of the islands in the region (except Magdalena Bay islands) [17,18]. For this study, our data sample includes 61 colonies of 19 seabird species: 5 Procellariiformes, 9 Charadriiformes, 4 Suliformes, and 1 Pelecaniformes, on ten islands and archipelagos (S1 Table).

For surface nesting-species (i.e., cormorants, pelicans, terns, and gulls), we surveyed active nests from land-based vantage points, complemented with surveys around the islands (boat counts), every 15 days during the whole breeding season. For burrow-nesting species (i.e., shearwaters, petrels, auklets, and murrelets), we conducted an exhaustive and intensive search of active burrows in all potential breeding sites. Burrow occupancy was determined either by recording apparently occupied burrows (i.e., with signs of activity such as guano, feathers, clear entrances, and footprints) or by directly confirming burrow content (i.e., adult, egg, or chick). On islands with accessible nesting sites and small size (i.e., Asunción and San Roque), we conducted a census (i.e., counted all available burrows) across the whole island, and either recorded apparently occupied burrows or checked burrow content using a hand-lamp or a burrowscope. Pairs nesting on artificial colonies installed on all the islands for social attraction [17] were included on our counts.

For those species with high nest density, such as the Western Gull (*Larus occidentalis*) on the Todos Santos Archipelago, Cassin’s Auklet (*Ptychoramphus aleuticus*) on San Jerónimo Island, Black-vented Shearwater (*Puffinus opisthomelas*) on Natividad and Guadalupe islands, Townsend’s Storm-Petrel (*Hydrobates socorroensis*) on Morro Prieto islet, and Guadalupe Murrelet (*Synthliboramphus hypoleucus*) on Zapato and Morro Prieto islets, we estimated nest or burrow densities during peak incubation by counting nests or burrows within circular or square plots that were randomly distributed and georeferenced [53,54]. We performed non-parametric resampling [55] of the density of either nests or burrows with apparent activity. This bootstrapping procedure does not include any assumptions about the data distribution. The algorithm we implemented to resample the data was proposed by [56]. We took a sample of *n* random observations with the possibility of replacement based on the original set of observed density data of burrows with activity, *σ*_obs_. We calculated the average for the bootstrapped version of the density, σ*, repeated the previous steps *B* = 2000 times, and obtained the bootstrapped distribution of the average nest and burrow density. With this, we obtained the 95% percentile bootstrap confidence interval (CI) for the average density of nests and burrows $\bar{\sigma^{*}}$. To calculate the total number of nests and burrows (*N*) per island, we multiplied the CI for the average density of nests and burrows $\bar{\sigma^{*}}$ by the nesting surface area *A*. The nesting surface area *A* was calculated using two different approaches: (1) based on an estimation done by field staff and confirmed every year on site. This only affected the Black-vented Shearwater on Natividad Island, the Cassin’s Auklet on San Jerónimo Island and the Western Gull on Todos Santos Island; (2) using the Gaussian kernel density estimation method, in which each georeferenced point contributes a Gaussian component to the total density. The result is a kernel density estimate derived entirely from the historical data (exhaustive searches) and serves as a non-parametric model of the distribution of nests and burrows. We defined the nesting area *A* as the 95% contour of the kernel density estimate. This only affected the Black-vented Shearwater on Guadalupe Island, the Townsend’s Storm-Petrel on Morro Prieto islet and the Guadalupe Murrelet on Zapato and Morro Prieto islets. In both cases, the field staff confirmed that the nesting area did not change and was the same throughout the breeding seasons of the data series. For most of the populations we did not need to estimate an area because we performed censuses instead of sampling.

### Population growth trends

#### Model and moving block bootstrap

We used the maximum number of breeding pairs for each nesting season on each island or archipelago to estimate the population growth rate (λ, lamda) for each of the 19 seabird species (S2 Table) using moving block bootstrap [57] and the relationship described in Eq (1):

$$N(t)=N_{0}\lambda^{t},$$ 1)

where *N*(*t*) is the number of breeding pairs in year *t*, *N*_*0*_ is the number of breeding pairs at the time of the first count of the period, and *λ* is the annual population growth rate. We tested that trends are approximately log-linear by plotting the yearly values of the logarithmic number of breeding pairs for each species at the different islands (Fig A in S1 Appendix). From the moving block bootstrap regression analysis, we were able to assess whether the seabird breeding populations are increasing, decreasing, or show no significant change (i.e., undetermined).

Moving block bootstrap makes no assumptions of the data distribution, and sampling is done with replacement [57]. We generated a bootstrap set N^*^ by randomly sampling *k* blocks of *l* = 3 consecutive records with replacement from our data set of maximum number of breeding pairs (S2 Table), *N*_*obs*_. We chose the number of blocks *k* so that *n*≈*k*×*l*, where *n* is the total number of records (i.e., monitored breeding seasons; e.g., *n* = 5 for Brandt’s Cormorant on Natividad Island) and *l* is the length of each block. We used moving block bootstrap to account for the autocorrelation in the time series [57]. We then calculated the bootstrap version λ^*^, of the population growth rate *λ*, by fitting Eq (1) to the bootstrap sample N^*^. We repeated these last steps *b* = 2000 times. After this, we calculated the 95% bootstrap percentile interval for λ^*^. Finally, we tested the following null hypotheses: increasing population, *H*_0_: *λ*≤1, *p*<*α* = 0.1 and decreasing population, *H*_0_: *λ*≥1, *p*<*α* = 0.1.

With results from Eq (1), we calculated the percent of change in the population using Eq (2):

$$\Delta N_{\%}=\frac{N(t)-N_{0}}{N_{0}}*100,$$ (2)

where *N*_*0*_ is the number of breeding pairs at the time of the first count of the period and *N(t)* is the number of breeding pairs in year *t*.

The regional population growth rate (*λ*_*R*_) and its percent of change were then calculated using Eqs (1) and (2), respectively. These population parameters were calculated for species with at least three consecutive years of data for all their colonies on different islands (18 of the 19 monitored species; Royal Tern was not included). We used the sum of the breeding numbers for all colonies for common years as the number of breeding pairs at the time *t*. For instance, for Brandt’s Cormorant we only have data for all its colonies in the period 2016–2018, although we have monitored some of its colonies since 2012 (e.g., Asunción and San Roque).

In order to summarize our findings, we performed a hierarchical bootstrap analysis [58] to calculate the average population growth rate of each seabird order (Charadriiformes, Pelecaniformes, Procellariiformes, and Suliformes). This analysis allowed us to account for the nested or multi-level structure of the data, as each species is associated with specific islands.

## Results

### Status of the seabird breeding populations

The BCPI current seabird assemblage is 23 species and a total of 129 colonies on 17 islands. Table 2 contains the most recent and updated information to date on the number of breeding pairs per seabird species per island on the BCPI. On average for the period 2014–2019, the BCPI supported ${400,000}_{-200,000}^{+300,000}$ breeding pairs at 61 colonies of 19 species (48% and 83% of the total, respectively). This means that considering just half the colonies (61 vs 129), at least between ca. 400,000 and 1.4 million individuals breed on this important seabird hotspot every year. San Benito, Natividad, and San Jerónimo are the top three islands in terms of abundance of breeding pairs (2014–2019 average): ${281,300}_{-280,600}^{+353,700},{101,400}_{-41,700}^{+34,000}$, and ${100,000}_{-76,600}^{+76,600}$, respectively.

**Table 2:** Breeding status of the seabird populations on the Baja California Pacific Islands for the 2017–2019 breeding seasons.

Name	Species	Coronado Norte	Coronado Sur	Terrón de Azúcar	Coronado Centro	Todos Santos Sur	Todos Santos Norte	San Martín	San Jerónimo	Guadalupe	Zapato	Morro Prieto	San Benito Oeste	San Benito Medio	San Benito Este	Natividad	San Roque	Asunción
Laysan Albatross	*Phoebastria immutabilis*									395^(e)^	745^(e)^	371^(e)^						
Black-vented Shearwater	*Puffinus opisthomelas*									E	229	400 (+120–110)	86	11	24	118,920 (+16,740 −16,290)		
Leach’s Storm-petrel	*Hydrobates leucorhous*	E		2									B	B	B			
Townsend’s Storm-petrel	*Hydrobates socorroensis*									2	11	890 (+520 -420)						
Ainley’s Storm-petrel	*Hydrobates cheimomnestes*									PB	2^(e)^	181^(e)^						
Ashy Storm-petrel	*Hydrobates homochroa*	5				20	55	PB										
Black Storm-petrel	*Hydrobates melania*	104^(a)^	PB	52^(a)^				PB					B	B	B			
Least Storm-petrel	*Hydrobates microsoma*												B	B	B			
Brown Pelican	*Pelecanus occidentalis*	597	442			723	E	376	166						78	531	37	243
Blue-footed Booby	*Sula nebouxii*								1									2^(g)^
Brown Booby	*Sula leucogaster*			13							27 (+8–8)							
Double-crested Cormorant	*Phalacrocorax auritus*	56	164			238	E	791	104						39	823	113	131
Brandt’s Cormorant	*Phalacrocorax penicillatus*	52	B	2	2	566	B	130	180		39 (+5–5)				22	2,400	4,747	3,228
Pelagic Cormorant	*Phalacrocorax pelagicus*	1	3	5		19	2		1									
Heermann’s Gull	*Larus heermanni*													121			42	
Western Gull	*Larus occidentalis*		194			5,990 (+1,100 −1,130)	2,470 (+820 −740)	1,382	2,442	B			B	568	442	300^(f)^	1,749	1,323
Caspian Tern	*Hydroprogne caspia*							186	18									
Elegant Tern	*Thalasseus elegans*								684								2,500	E
Royal Tern	*Thalasseus maximus*								171								870	
Scripps’s Murrelet	*Synthliboramphus scrippsi*	25^(a)^	34^(a)^	27^(a)^	11^(a)^	84	17	B	3				145	7	18			
Guadalupe Murrelet	*Synthliboramphus hypoleucus*									275^(e)^	2,360 (+530–500)	1,600 (+480–420)	2	1				
Craveri’s Murrelet	*Synthliboramphus craveri*							1^(b)^					1			1	5	1
Cassin’s Auklet	*Ptychoramphus aleuticus*	1^(h)^	20	B		30	33	B^(c)^	218,250^(d)^ (+17200 −17410)	E		202	472,606 (+62,443 -62,473	7,621	79,224 (+13,190 -12,541)	12	1,659	2,602
**Total breeding taxa**		**8**	**8**	**7**	**2**	**8**	**6**	**10**	**11**	**5**	**7**	**6**	**9**	**9**	**10**	**7**	**9**	**7**

Records show the maximum number of breeding pairs per species per island during our 2017–2019 surveys. B: Breeder; PB: Probable breeder; E: Extirpated.

^(a)^ Maximum number recorded but no exhaustive survey was possible.

^(b)^ Record by [59] for the 2007–2008 breeding season, during our own surveys we just found and confirmed Scripps’s Murrelet.

^(c)^ 136 apparently occupied nests were found in 2017 but no exhaustive survey was possible.

^(d)^ Data from 2016.

^(e)^ Data from 2020.

^(f)^ Data from 2015.

^(g)^ New record.

^(h)^ Previously considered extirpated.

The most widespread species is Cassin’s Auklet with 14 colonies, followed by Brandt’s Cormorant with 13, Western Gull with 12 and Scripps’s Murrelet with 11. The less widespread species are Blue-footed Booby, Brown Booby, Caspian Tern, Elegant Tern, Hermann’s Gull and Royal Tern with two colonies each (Fig B in S1 Appendix).

The San Benito Archipelago hosts the greatest seabird assemblage with 13 breeding species, followed by the Coronado Archipelago and San Jerónimo Island (11 species), and San Martín Island (10 species) (Fig C in S1 Appendix). It stands-out the number of species on San Jerónimo and San Martín despite their relatively small size (48 and 265 hectares, respectively). We are reporting one new record, the Blue-footed Booby on Asunción Island, and one new recolonization of a previously extirpated species, the Cassin’s Auklet on Coronado Norte (Table 2).

The island with the lowest number of breeding species is Guadalupe (5 species), despite being the biggest (24,171 ha) although the most oceanic island (Figs C and D in S1 Appendix). Two species are extirpated from this island due to predation by feral cats: Black-vented Shearwater and Cassin’s Auklet, which are restricted to the nearby cat-free Zapato and Morro Prieto islets (Table 2).

### Seabird population trends

**Fig 2:**
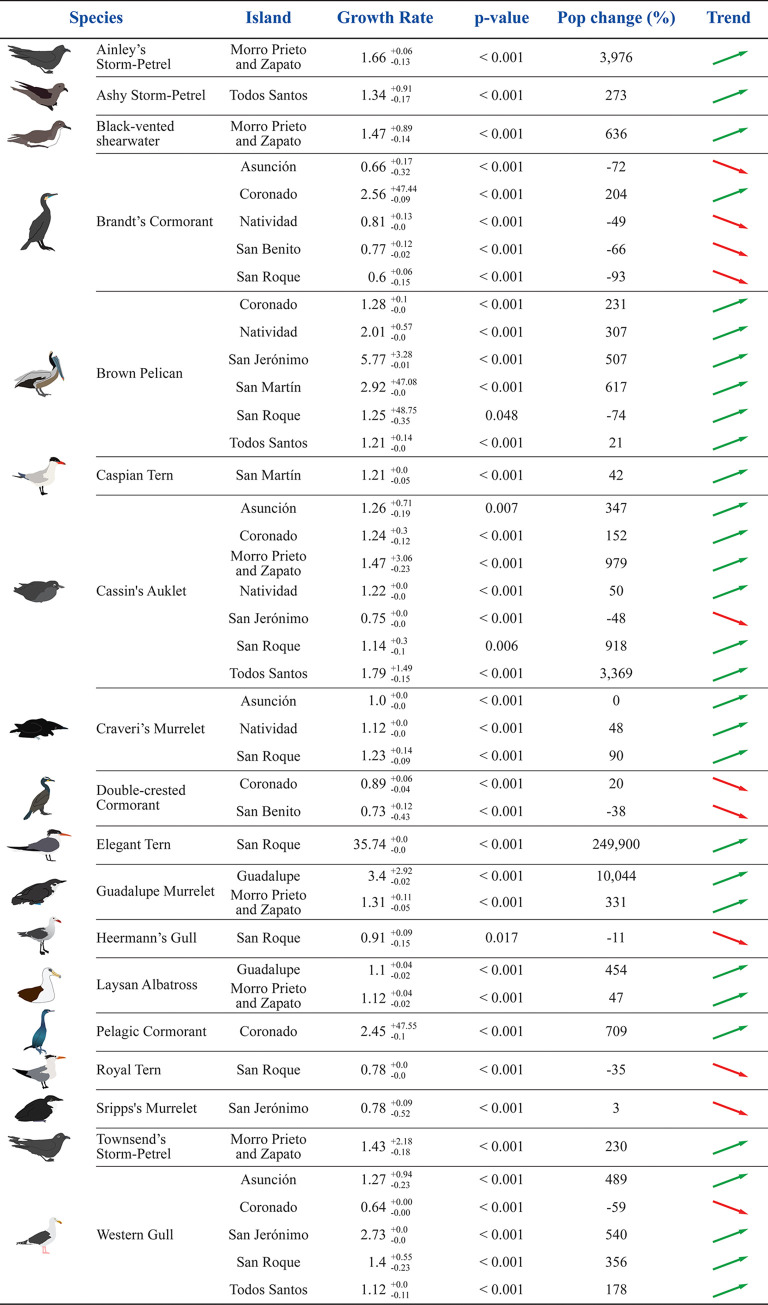
Seabird population trends on the Baja California Pacific Islands for the period 2014–2019. Only species colonies that tested the following null hypotheses: Increasing population, *H*_0_: *λ*≤1, *p*<*α* = 0.1 (31 colonies, 14 species) and decreasing population, *H*_0_: *λ*≥1, *p*<*α* = 0.1 (11 colonies, 7 species) are shown.

**Fig 3:**
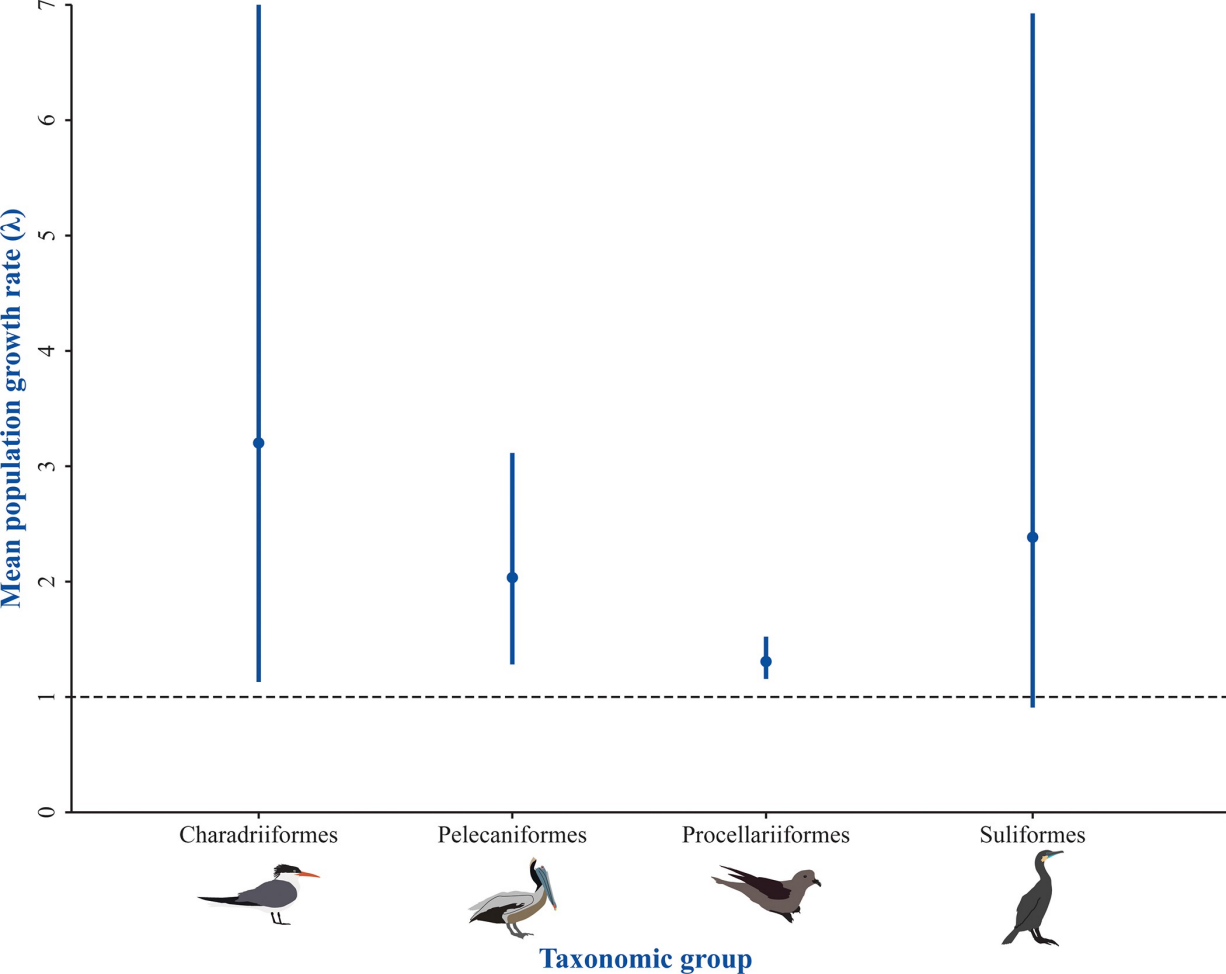
Population trends per taxonomic group. Points represent the hierarchical bootstrapped means from the medians of the population growth rates (λ) shown in S1 Table. Bars represent the 95% bootstrap interval. The horizontal dashed line shows λ = 1 which indicates no population change.

We were able to assess the population growth rates of 61 colonies of 19 species of the BCPI. Estimated population growth rates and the percent of change of the populations over the monitored timeframes are shown in S1 Table. Thirty-one (50.8%) populations of 14 species are significantly increasing while eleven (18.0%) populations of seven species are decreasing (Fig 2 and Table 3). We did not find statistical significance for 19 (31.1%) populations thus were unable to determine an increasing nor decreasing trend. We also found that all taxonomic groups evaluated are growing, and that populations of murrelets, auklets, gulls, and terns (Charadriiformes) show the fastest growth (Fig 3). The median growth rate for all seabird populations in the BCPI was λ = 1.19.

**Table 3:** Summary of population trends for 61 colonies of 19 seabird species on 10 islands and archipelagos in the Mexican Pacific off the Baja California Peninsula.

Island/Archipelago	No. Populations	Population trend status		
		Increasing	Decreasing	Undetermined
Coronado	7	4	2	1
Todos Santos	8	4	0	4
San Martín	5	2	0	3
San Jerónimo	8	2	2	4
Guadalupe	2	2	0	0
Morro Prieto and Zapato	6	6	0	0
San Benito	4	0	2	2
Natividad	6	3	1	2
San Roque	9	5	3	1
Asunción	6	3	1	2
**TOTAL**	**61**	**31**	**11**	**19**
**%**	**100**	**51**	**18**	**31**

Cassin’s Auklet and Brown Pelican are the species that have the most populations—six each—with a positive growing trend, while Brandt’s Cormorant has the most decreasing populations with four colonies. Three species stand out for their fast population growth rates: Elegant Tern on San Roque Island ($35.74_{-0.0}^{+0.0}$; 249,900% population change 2017–2019), Brown Pelican on San Jerónimo Island ($5.77_{-0.01}^{+3.28}$; 507% population change 2015–2017), and Guadalupe Murrelet on Guadalupe Island ($3.4_{-0.02}^{+2.92}$; 10,044% population change 2015–2019) (Fig 2 and S1 Table).

**Fig 4:**
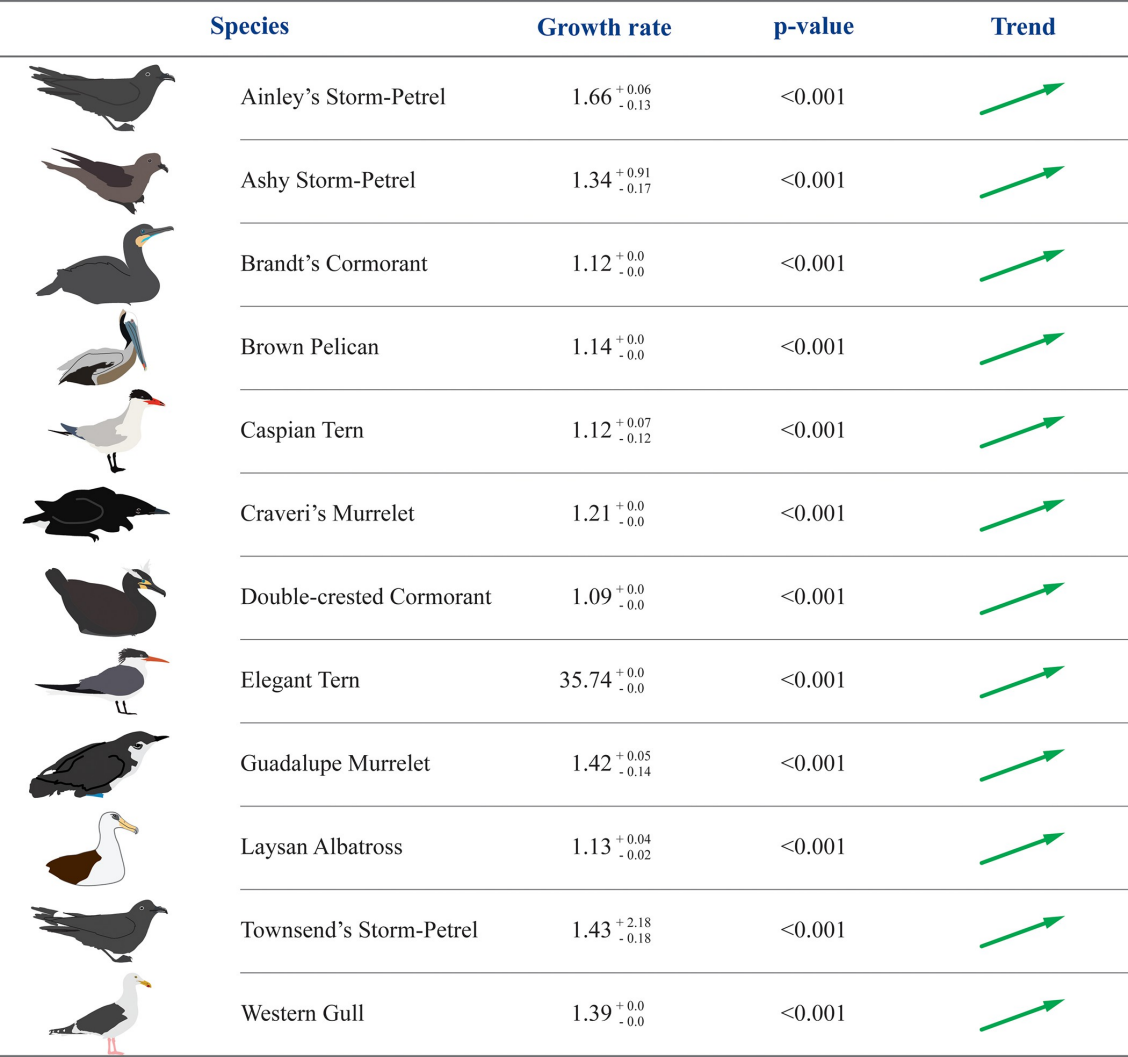
Seabirds with a positive regional population trend on the Baja California Pacific Islands for the period 2014–2019. For an increasing population, the following null hypothesis was tested: *H*_0_: *λ*≤1, *p*<*α* = 0.1.

For the period 2014–2019, twelve (66.7%) of the 18 species for which we estimated a regional population trend are significantly increasing, including seven surface-nesting species (Brandt’s Cormorant, Brown Pelican, Caspian Tern, Double-crested Cormorant, Elegant Tern, Laysan Albatross and Western Gull) and five burrow-nesting species (Ainley’s, Ashy and Townsend’s Storm-Petrels, and Craveri’s and Guadalupe Murrelets). Four species are restricted to the remote Guadalupe, Morro Prieto and Zapato: Ainley’s and Townsend’s Storm-Petrels, Guadalupe Murrelet and Laysan Albatross (Fig 4). Other two species (Black-vented Shearwater and Pelagic Cormorant) have a population growth rate *λ*>1, which suggests a positive growth despite not statistically significant. This suggests that 78% (14) of the assessed species (n = 18) on the BCPI show a positive regional growth trend.

## Discussion

We provide the most comprehensive and recent breeding status of this region’s seabirds, including the first multi-colony and multi-species evaluations of population growth trends. Seabird populations in the BCPI have been showing an improvement both in number of species, colonies and breeding pairs as has been demonstrated by [17] and our own present work. Our estimation of ca. 400,000–1,400,000 breeding individuals is like the estimates by [11] from aerial at-sea and coastal surveys for seabirds off southern California (from Cambria, California, USA, to the Mexican border) in 1999–2002, and below the estimate of 2.4 million breeding pairs for the BCPI region done by [13] from a literature review and censuses in 1999–2003. We argue this is not because the seabird abundance has decreased in the region but more to the fact of how [13] calculated such figure from bibliographical population estimates combined with few population censuses between 1999 and 2003. Also, we consider our estimation conservative since our analyses only considered nearly half (61) of the 129 colonies that currently occur within the BCPI, and we are not including breeding numbers from colonies on the islands of Magdalena Bay (i.e., Santa Magdalena, Santa Margarita and Creciente).

The San Benito Archipelago, Natividad and San Jerónimo remain to be the three islands with the greatest abundance, with the former and the latter hosting the greatest number of breeding seabird species of all the BCPI. The case of San Jerónimo stands out given that it doubled its species richness in a couple of decades, from four species reported by [13] to eleven species at present time (Table 2). This reveals the importance of this small island for the seabirds in the BCPI and thus the relevance to prevent and mitigate threats such as guano mining—still a latent danger since there exists an active lease to exploit it although it has been demonstrated that seabird guano is more valuable if retain in the ecosystem for aiding in nutrient deposition from the marine to the terrestrial environment [60]—and the introduction of invasive alien species both of which severely affected the seabird populations on this island for many decades. This increase in species might be related to the fact that the island is free from invasive mammals since 1999 [18] and that it has been protected since 2016 as part of the Baja California Pacific Islands Biosphere Reserve [22]. But also, to the existence since 2014 of community-led marine reserves to protect habitat and restore populations of abalone (*Haliotis spp*.) as well as to increase fish recruitment, which have already proven to have an indirect positive effect on marine mammals such as the California Sea Lion (*Zalophus californianus*) [61], plus the fact that San Jerónimo lies within an upwelling center making its surrounding environment highly productive [62].

Specie richness not only remained high on the BCPI but it increased during the past couple of decades, with the number of colonies increasing twofold, from 62 [13] to 129—partly because we surveyed all islands and islets and provide disaggregated information for each island within archipelagos in Table 2—, and with the number of species increasing from 19 [13] to 23.

No previous evaluation of the trends of this seabird populations existed, particularly at a regional scale. We found that 46 out of 61 seabird populations on the BCPI are growing, which means that a third of all known breeding populations (N = 129) have a positive population growth trend. In contrast, 15 out of 61 seabird populations are declining, which represents just 12% of the whole breeding populations. The median growth rate of λ = 1.19 for all assessed seabird populations is similar to that found by [32] of λ = 1.119 for 181 seabird populations of 69 species on islands around the world. It is also similar to the population growth rate recorded by [24], λ = 1.10, and [63], λ = 1.35, for a steadily expanding Laysan Albatross population on Guadalupe Island. Currently, this breeding population has a λ = 1.1, just barely higher than almost a decade ago, and it remains to be higher than λ observed for other species of albatross [64].

We found a high, atypical λ = 35.74 for the Elegant Tern on San Roque Island. This is explained because it is a newly established colony where the species recolonized the island in 2017 with one breeding pair, this after a decade of a systematic seabird social attraction program [17]. For the following years, the colony has significantly increased in breeding numbers, which is must probably the result of immigration playing an important role in the formation of the new colony [32]. A contrasting example on this same island is that of the Royal Tern that had been extirpated for almost a century, with a λ = 0.78 despite also being a newly formed colony in the same year as that of Elegant Tern [17]. For this species we suggest that the decreasing trend is mostly related to the short timeframe since the colony formation and thus would suggest keeping monitoring further years to reevaluate such trend. It might also be related to what [32] point out about growth rate declining over time because immigrant birds become a smaller fraction of the breeding population. The results obtained for this tern species can also be related to the low philopatry known for gulls and terns, where local population dynamics are influenced by emigration and immigration processes that can produce annual variations in breeding numbers [1]. Environmental factors have also an influence in the distribution of Elegant Tern since it has been proved that this species adapts to changing oceanographic conditions and fish availability, which makes it migrate from the Gulf of California to Southern California searching more productive waters [65].

At a regional scale, 14 out of 23 species on the BCPI show growing populations. It stands out that four of these species occur on the Guadalupe-Morro Prieto/Zapato cluster. This is not surprising because the islets are seabird havens where no pressing threats exist and no perturbations such as the existence of invasive mammals have ever occurred, and Guadalupe Island has been subject to a comprehensive and long-term restoration program, which has had a positive effect on the Laysan Albatross population [52] and very recently on the Guadalupe Murrelet thanks to the protection of its potential nesting habitat from the presence of feral cats with an exclusion fence since 2014 and with the four-year eradication program that is being carried out on the island. The case of the Guadalupe Murrelet can be explained as a rapid population growth rate following a successful invasive mammal eradication by immigration from the nearby islets [32].

The BCPI region has been subject to variable and extreme environmental conditions that are known to negatively affect seabird populations. The “Blob” occurred in 2013–2015 and reached the coasts of the Baja California Peninsula in May 2014, lasting until April 2015 [66]. It was followed by strong ENSO conditions, “Godzilla”, that lasted until the end of 2016 [67]. This extreme conditions severely affected the CCS and its species, including massive die-offs of seabirds, such as Cassin’s Auklets in the central CCS in the western coast of the United States [68]. For the BCPI region, the 2014–2016 marine heatwaves have been the most intense and persistent events recorded to date [69]. For instance, we recorded a 38% and 50% nest abandonment for the Brown Pelican and Brandt’s Cormorant, respectively, in 2015, between the “Blob” and “Godzilla” marine heatwaves. Nonetheless, despite these severe environmental conditions in recent years, the seabird breeding abundances and population growth rates that we report here indicate that the BCPI seabird populations are resilient to environmental variations [70]. Such resilience has and can be further strengthened from conservation actions such as the eradication of invasive mammals [18,23] and social attraction techniques [17].

## Supporting information

S1 Table Population trends for 19 seabird species at their colonies on 10 islands/archipelagos in the Mexican Pacific off the Baja California Peninsula. A 95% bootstrap interval was calculated for population growth rates **(***λ***)** using moving block bootstrap; central value, lower and upper limits are given. Percent change was estimated using Eq (2) for the period described. ^**a**^Island/archipelago ordered North to South. ^**b**^N indicates the most recent count of breeding pairs with the year in parentheses. ^c^Increasing (+); Decreasing (-); Not Determined (ND).
(XLSX)

S2 Table Database of maximum number of breeding pairs used to estimate the population growth rates for seabirds on the Baja California Pacific Islands. (XLSX)

S1 Appendix Seabird population trends Mexican Pacific Islands. (PDF)

## References

[1] Oro D, Martínez-Abraín A. Ecology and Behaviour of Seabirds. In: Duarte CM, Helgueras AL, editors. Marine Ecology. Encyclopedia of Life Support Systems. Oxford, UK: Eolss Publishers Co. Ltd. / UNESCO; 2009.

[2] Croxall JP, Butchart SHM, Lascelles B, Stattersfield AJ, Sullivan B, Symes A, et al. Seabird conservation status, threats and priority actions: a global assessment. Bird Conservation International. 2012;22(01):1–34. doi: 10.1017/S0959270912000020

[3] Paleczny M, Hammill E, Karpouzi V, Pauly D. Population Trend of the World’s Monitored Seabirds, 1950–2010. PLoS ONE. 2015;10(6):e0129342. doi: 10.1371/journal.pone.0129342 26058068 PMC4461279

[4] Votier SC, Sherley RB. Seabirds. Current Biology. 2017;27(11):R448–R50. doi: 10.1016/j.cub.2017.01.042 28586675

[5] Dias MP, Martin R, Pearmain EJ, Burfield IJ, Small C, Phillips RA, et al. Threats to seabirds: A global assessment. Biological Conservation. 2019;237:525–37. doi: 10.1016/j.biocon.2019.06.033

[6] NABCI. The State of North America’s Birds. Ottawa, Ontario, Canada: North American Bird Conservation Initiative. Environment and Climate Change Canada, 2016.

[7] Oedekoven CS, Ainley DG, Spear LB. Variable responses of seabirds to change in marine climate: California Current, 1985–1994. Marine Ecology Progress Series. 2001;212:265–81.

[8] Sydeman WJ, Mills KL, Santora JA, Thompson SA, Bertram DF, Morgan KH, et al. Seabirds and climate in the California Current-a synthesis of change. California Cooperative Oceanic Fisheries Investigations Reports. 2009;50:82–104.

[9] Ainley DG, Hyrenbach KD. Top-down and bottom-up factors affecting seabird population trends in the California current system (1985–2006). Progress in Oceanography. 2010;84(3–4):242–54. doi: 10.1016/j.pocean.2009.10.001

[10] Nur N, Jahncke J, Herzog MP, Howar J, Hyrenbach KD, Zamon JE, et al. Where the wild things are: Predicting hotspots of seabird aggregations in the California Current System. Ecological Applications. 2011;21(6):2241–57. doi: 10.1890/10-1460.1 21939058

[11] Mason JW, McChesney GJ, McIver WR, Carter HR, Takekawa JY, Golightly RT, et al. At-sea distribution and abundance of seabirds off southern California: A 20-year comparison. Studies in Avian Biology 2007. p. 1–101.

[12] Gaston AJ, Bertram DF, Boyne AW, Chardine JW, Davoren G, Diamond AW, et al. Changes in Canadian seabird populations and ecology since 1970 in relation to changes in oceanography and food webs. Environmental Reviews. 2009;17:267–86. doi: 10.1139/a09-013

[13] Wolf S, Keitt B, Aguirre-Muñoz A, Tershy B, Palacios E, Croll D. Transboundary seabird conservation in an important North American marine ecoregion. Environmental Conservation. 2006;33(04):294–305. doi: 10.1017/S0376892906003353

[14] Berlanga H, Rodríguez-Contreras V, Oliveras de Ita A, Escobar M, Rodríguez L, Vieyra J, et al. Red de Conocimientos sobre las Aves de México (AVESMX): Comisión Nacional para el Conocimiento y Uso de la Biodiversidad; 2008. Available from: http://avesmx.conabio.gob.mx/.

[15] SEMARNAT. Programa de Acción para la Conservación de las Especies Aves Marinas. Ciudad de México, México: Secretaría de Medio Ambiente y Recursos Naturales, Comisión Nacional de Áreas Naturales Protegidas y Comisión Nacional para el Conocimiento y Uso de la Biodiversidad; 2021.

[16] González-García F, Gómez de Silva H. Especies endémicas: riqueza, patrones de distribución y retos para su conservación. In: Gómez de Silva H, Oliveras de Ita A, editors. Conservación de Aves: Experiencias en México. México, D.F.: CIPAMEX; 2003. p. 150–94.

[17] Bedolla-Guzmán Y, Méndez-Sánchez F, Aguirre-Muñoz A, Félix-Lizárraga M, Fabila-Blanco A, Bravo-Hernández E, et al. Recovery and current status of seabirds on the Baja California Pacific Islands, Mexico, following restoration actions. In: Veitch CR, Clout MN, Martin AR, Russell JC, West CJ, editors. Island invasives: scaling up to meet the challenge. Occasional Paper SSC no. 62. Gland, Switzerland: IUCN; 2019. p. 531–8.

[18] Aguirre-Muñoz A, Bedolla-Guzmán Y, Hernández-Montoya J, Latofski-Robles M, Luna-Mendoza L, Méndez-Sánchez F, et al. The Conservation and Restoration of the Mexican Islands, a Successful Comprehensive and Collaborative Approach Relevant for Global Biodiversity. In: Ortega-Rubio A, editor. Mexican Natural Resources Management and Biodiversity Conservation: Recent Case Studies. Cham: Springer International Publishing; 2018. p. 177–92.

[19] McChesney GJ, Tershy BR. History and Status of Introduced Mammals and Impacts to Breeding Seabirds on the California Channel and Northwestern Baja California Islands. Colonial Waterbirds. 1998;21(3):335–47. doi: 10.2307/1521646

[20] Everett WT, Anderson DW. Status and conservation of the breeding seabirds of the offshore Pacific islands of Baja California and the Gulf of California. Cambridge, UK: International Council for Bird Preservation, 1991.

[21] Aguirre-Muñoz A, Croll DA, Donlan CJ, Henry RW, Hermosillo MA, Howald GR, et al. High-impact Conservation: Invasive Mammal Eradications from the Islands of Western México. AMBIO. 2008;37(2):101–7. doi: 10.1579/0044-7447(2008)37[101:hcimef]2.0.co;2 18488552

[22] Aguirre-Muñoz A, Méndez-Sánchez F. The New Baja California Pacific Islands Biosphere Reserve Sets a Conservation Benchmark: All Mexican Islands are Now Protected. FREMONTIA Journal of the California Native Plant Society [Internet]. 2017; 42(3):[27–31 pp.]. Available from: https://www.cnps.org/wp-content/uploads/2018/06/V45_N3_Islands_Fremontia-English_FINAL_web.pdf.

[23] Aguirre-Muñoz A, Samaniego-Herrera A, Luna-Mendoza L, Ortiz-Alcaraz A, Rodríguez-Malagón M, Méndez-Sánchez F, et al. Island restoration in Mexico: ecological outcomes after systematic eradications of invasive mammals. In: Veitch CR, Clout MN, Towns DR, editors. Island Invasives: Eradication and Management Proceedings of the International Conference on Island Invasives. Occasional Paper of the IUCN Species Survival Commission No. 42. Gland, Switzerland and Auckland, New Zealand: IUCN and CBB; 2011. p. 250–8.

[24] Hernández-Montoya JC, Luna-Mendoza L, Aguirre-Muñoz A, Méndez-Sánchez F, Félix-Lizárraga M, Barredo-Barberena JM. Laysan Albatross on Guadalupe Island, México: current status and conservation actions. Monographs of the Western North American Naturalist. 2014;7:543–54.

[25] Bedolla-Guzmán Y, Masello JF, Aguirre-Muñoz A, Lavaniegos BE, Quillfeldt P. Breeding biology, chick growth, and diet of the Least Storm-Petrel *Oceanodroma microsoma* on Islas San Benito, Mexico. Marine Ornithology. 2017;45(2):129–38.

[26] Graham NAJ, Wilson SK, Carr P, Hoey AS, Jennings S, MacNeil MA. Seabirds enhance coral reef productivity and functioning in the absence of invasive rats. Nature. 2018;559(7713):250–3. doi: 10.1038/s41586-018-0202-3 29995864

[27] Jones HP, Holmes ND, Butchart SHM, Tershy BR, Kappes PJ, Corkery I, et al. Invasive mammal eradication on islands results in substantial conservation gains. Proceedings of the National Academy of Sciences. 2016;113(15):4033–8. doi: 10.1073/pnas.1521179113 27001852 PMC4839448

[28] Towns DR. Eradications of vertebrate pests from islands around New Zealand: what have we delivered and what have we learned? In: Veitch CR, Clout MN, Towns DR, editors. Island Invasives: Eradication and Management Proceedings of the International Conference on Island Invasives. Occasional Paper of the IUCN Species Survival Commission No. 42. Gland, Switzerland and Auckland, New Zealand: IUCN and CBB; 2011. p. 364–71.

[29] Towns DR, West CJ, Broome KG. Purposes, outcomes and challenges of eradicating mammals from New Zealand islands: an historical perspective. Wildlife Research. 2013;40:94–107.

[30] Buxton RT, Jones C, Moller H, Towns DR. Drivers of Seabird Population Recovery on New Zealand Islands after Predator Eradication. Conservation Biology. 2014;28(2):333–44. doi: 10.1111/cobi.12228 24527858

[31] Borrelle SB, Boersch-Supan PH, Gaskin CP, Towns DR. Influences on recovery of seabirds on islands where invasive predators have been eradicated, with a focus on Procellariiformes. Oryx. 2016:1–13. Epub 2016/12/19. doi: 10.1017/s0030605316000880

[32] Brooke MdL, Bonnaud E, Dilley BJ, Flint EN, Holmes ND, Jones HP, et al. Seabird population changes following mammal eradications on islands. Animal Conservation. 2018;21(1):3–12. doi: 10.1111/acv.12344

[33] Spatz DR, Holmes ND, Will DJ, Hein S, Carter ZT, Fewster RM, et al. The global contribution of invasive vertebrate eradication as a key island restoration tool. Scientific Reports. 2022;12(1):13391. doi: 10.1038/s41598-022-14982-5 35948555 PMC9365850

[34] Aguirre Muñoz A, Samaniego Herrera A, Luna Mendoza L, Ortiz Alcaraz A, Méndez Sánchez F, Hernández Montoya J. La restauración ambiental exitosa de las islas de México: una reflexión sobre los avances a la fecha y los retos por venir. In: Ceccon E, Martínez-Garza C, editors. Experiencias mexicanas en la restauración de los ecosistemas. Cuernavaca, Morelos, México: Universidad Nacional Autónoma de México, Centro Regional de Investigaciones Multidisciplinarias, Universidad Autónoma del Estado de Morelos, Comisión Nacional para el Conocimiento y Uso de la Biodiversidad; 2016. p. 487–512.

[35] Towns DR. Understanding seabird responses to invasive mammal eradications from islands needs systematic monitoring. Animal Conservation. 2018;21(1):15–6. doi: 10.1111/acv.12391

[36] Ortiz-Alcaraz A, Aguirre-Muñoz A, Méndez-Sánchez F, Rojas-Mayoral E, Solís-Carlos F, Rojas-Mayoral B, et al. Ecological restoration of Socorro Island, Revillagigedo Archipelago, Mexico: the eradication of feral sheep and cats. In: Veitch CR, Clout MN, Martin AR, Russell JC, West CJ, editors. Island invasives: scaling up to meet the challenge. Occasional Paper SSC no. 62. Gland, Switzerland: IUCN; 2019. p. 267–73.

[37] Jones HP, Kress SW. A Review of the World’s Active Seabird Restoration Projects. Journal of Wildlife Management. 2012;76(1):2–9. doi: 10.1002/jwmg.240

[38] Kappes P, Jones H. Integrating seabird restoration and mammal eradication programs on islands to maximize conservation gains. Biodivers Conserv. 2014;23(2):503–9. doi: 10.1007/s10531-013-0608-z

[39] Pacific Rim Conservation, National Audubon Society, The Nature Conservancy, New Zealand Department of Conservation, Museum of New Zealand Te Papa Tongarewa, Northern Illinois University. The Seabird Restoration Database 2021 [cited 2021 18 September]. Available from: https://www.seabirddatabase.org/.

[40] Albores-Barajas YV, Soldatini C, Ramos-Rodríguez A, Alcala-Santoyo JE, Carmona R, Dell’Omo G. A new use of technology to solve an old problem: Estimating the population size of a burrow nesting seabird. PLoS ONE. 2018;13(9):e0202094. doi: 10.1371/journal.pone.0202094 30216342 PMC6157828

[41] Whitworth DL, Carter HR, Palacios E, Gress F. Breeding of Craveri’s Murrelet *Synthliboramphus craveri* at four islands off west-central baja california, México. Marine Ornithology. 2018;46(2):117–24.

[42] Whitworth DL, Carter HR, Palacios E, Gress F. At-sea congregation surveys to assess the status of Scripps’s Murrelets *Synthliboramphus scrippsi* at islands off western Baja California, México. Marine Ornithology. 2020;48(1):41–52.

[43] Whitworth DL, Carter HR, Palacios E, Koepke JS, McIver WR, Hamilton CD, et al. The rarest alcid: status and history of the Guadalupe Murrelet *Synthliboramphus hypoleucus* at Isla Guadalupe, Mexico (1892–2007). Marine Ornithology. 2021;49:133–43.

[44] Buckley PA, Downer R. Modelling Metapopulation Dynamics for Single Species of Seabirds. In: McCullough DR, Barrett RH, editors. Wildlife 2001: Populations. Dordrecht: Springer Netherlands; 1992. p. 563–85.

[45] Munilla I, Genovart M, Paiva VH, Velando A. Colony Foundation in an Oceanic Seabird. PLoS ONE. 2016;11(2):e0147222. doi: 10.1371/journal.pone.0147222 26909694 PMC4766187

[46] UNEP-WCMC. Protected Area Profile for Mexico from the World Database of Protected Areas, July 2020. 2020 [12 July 2020]. Available from: www.protectedplanet.net.

[47] Latofski-Robles M, Aguirre-Muñoz A, Méndez-Sánchez F, Reyes-Hernández H, Schlüter S. Prioritazing restorarion actions for the islands of México. Monographs of the Western North American Naturalist. 2014;7:435–41.

[48] Vidal RM, Berlanga H, Del Coro Arizmendi M. Important Bird Areas: Mexico. 2009. In: Important Bird Areas Americas—Priority sites for biodiversity conservation [Internet]. Quito, Ecuador: BirdLife International (BirdLife Conservation Series No. 16).

[49] Samaniego Herrera A, Peralta García A, Aguirre Muñoz A, editors. Vertebrados de las islas del Pacífico de Baja California: Guía de Campo. México, D.F.: Grupo de Ecología y Conservación de Islas, A.C.; 2007.

[50] Luna-Mendoza L, Aguirre-Muñoz A, Hernández-Montoya J, Torres-Aguilar M, García-Carreón JS, Puebla-Hernández O, et al. Ten years after feral goat eradication: the active restoration of plant communities on Guadalupe Island, Mexico. In: Veitch CR, Clout MN, Martin AR, Russell JC, West CJ, editors. Island invasives: scaling up to meet the challenge. Occasional Paper SSC no. 62. Gland, Switzerland: IUCN; 2019. p. 571–5.

[51] Sydeman W, Elliott M. Developing the California Current Integrated Ecosystem Assessment, Module I: Select Time-Series of Ecosystem State. 2008.

[52] Hernández Montoya JC, Juárez-Rodríguez M, Méndez-Sánchez F, Aguirre-Muñoz A, Rojas-Mayoral E, Íñigo-Elias E, et al. Sexual Dimorphism and Foraging Trips of the Laysan Albatross (*Phoebastria immutabilis*) on Guadalupe Island. Animals. 2019;9(6):364. doi: 10.3390/ani9060364 31212935 PMC6617548

[53] Keitt BS, Tershy BR, Croll DA. Breeding biology and conservation of the Black-vented Shearwater Puffinus opisthomelas. Ibis. 2003;145(4):673–80. doi: 10.1046/j.1474-919X.2003.00223.x

[54] Parker GC, Rexer-Huber K. Guidelines for designing population surveys of burrowing petrels. Agreement on the Conservation of Albatrosses and Petrels, 2020.

[55] Berrar D. Introduction to the Non-Parametric Bootstrap. In: Ranganathan S, Gribskov M, Nakai K, Schönbach C, editors. Encyclopedia of Bioinformatics and Computational Biology. Oxford: Academic Press; 2019. p. 766–73.

[56] Carpenter J, Bithell J. Bootstrap confidence intervals: when, which, what? A practical guide for medical statisticians. Statistics in Medicine. 2000;19(9):1141–64. 10.1002/(SICI)1097-0258(20000515)19:9<1141::AID-SIM479>3.0.CO;2-F. 10797513

[57] Efron B, Tibshirani RJ. An Introduction to the Bootstrap: Chapman and Hall/CRC; 1994.

[58] Saravanan V, Berman GJ, Sober SJ. Application of the hierarchical bootstrap to multi-level data in neuroscience. Neurons, behavior, data analysis and theory. 2020;3(5). Epub 2020/01/01. ; PubMed Central PMCID: PMC7906290. 33644783 PMC7906290

[59] Whitworth DL, Carter HR, Parker MW, Gress F, Booker M. Long-Term Monitoring of Scripps’s Murrelet and Guadalupe Murrelet at San Clemente Island, California: Evaluation of Baseline Data in 2012–2016. Western North American Naturalist. 2018;78(3):457–73, 17.

[60] Plazas-Jiménez D, Cianciaruso MV. Valuing Ecosystem Services Can Help to Save Seabirds. Trends in Ecology & Evolution. 2020;35(9):757–62. doi: 10.1016/j.tree.2020.06.008 32773149

[61] Arias-Del-Razo A, Schramm Y, Heckel G, Sáenz-Arroyo A, Hernández A, Vázquez L, et al. Do marine reserves increase prey for California sea lions and Pacific harbor seals? PLoS ONE. 2019;14(6):e0218651. doi: 10.1371/journal.pone.0218651 31220168 PMC6586349

[62] Woodson CB, Micheli F, Boch C, Al-Najjar M, Espinoza A, Hernandez A, et al. Harnessing marine microclimates for climate change adaptation and marine conservation. Conservation Letters. 2019;12(2):e12609. 10.1111/conl.12609.

[63] Gallo-Reynoso JP, Figueroa-Carranza AL. The breeding colony of Laysan albatrosses on Isla de Guadalupe, Mexico. Western Birds. 1996;27:70–6.

[64] Weimerskirch H, Delord K, Barbraud C, Le Bouard F, Ryan PG, Fretwell P, et al. Status and trends of albatrosses in the French Southern Territories, Western Indian Ocean. Polar Biol. 2018;41(10):1963–72. doi: 10.1007/s00300-018-2335-0

[65] Velarde E, Ezcurra E, Horn MH, Patton RT. Warm oceanographic anomalies and fishing pressure drive seabird nesting north. Science Advances. 2015;1(5):e1400210. doi: 10.1126/sciadv.1400210 26601193 PMC4640602

[66] Bond NA, Cronin MF, Freeland H, Mantua N. Causes and impacts of the 2014 warm anomaly in the NE Pacific. Geophysical Research Letters. 2015;42(9):3414–20. 10.1002/2015GL063306.

[67] Di Lorenzo E, Mantua N. Multi-year persistence of the 2014/15 North Pacific marine heatwave. Nature Climate Change. 2016;6(11):1042–7. doi: 10.1038/nclimate3082

[68] Cavole LM, Demko AM, Diner RE, Giddings A, Koester I, Pagniello CMLS, et al. Biological Impacts of the 2013–2015 Warm-Water Anomaly in the Northeast Pacific. Winners, Losers, and the Future. Oceanography. 2016;29(2):273–85.

[69] Arafeh-Dalmau N, Montaño-Moctezuma G, Martínez JA, Beas-Luna R, Schoeman DS, Torres-Moye G. Extreme Marine Heatwaves Alter Kelp Forest Community Near Its Equatorward Distribution Limit. Frontiers in Marine Science. 2019;6(499). doi: 10.3389/fmars.2019.00499

[70] Velarde E, Ezcurra E. Are seabirds’ life history traits maladaptive under present oceanographic variability? The case of Heermann’s Gull (Larus heermanni). The Condor. 2018;120(2):388–401. doi: 10.1650/condor-17-5.1

