# Supplementary material for: Population trends of seabirds in Mexican Islands at the California Current System
Source: PLoS One. 2022 Oct 7;17(10):e0258632. doi: 10.1371/journal.pone.0258632 (PMC9543960; doi:10.1371/journal.pone.0258632)
Supplement: S1 Appendix — (PDF) [file pone.0258632.s003.pdf]

S1 APPENDIX. SEABIRD POPULATION TRENDS MEXICAN PACIFIC ISLANDS

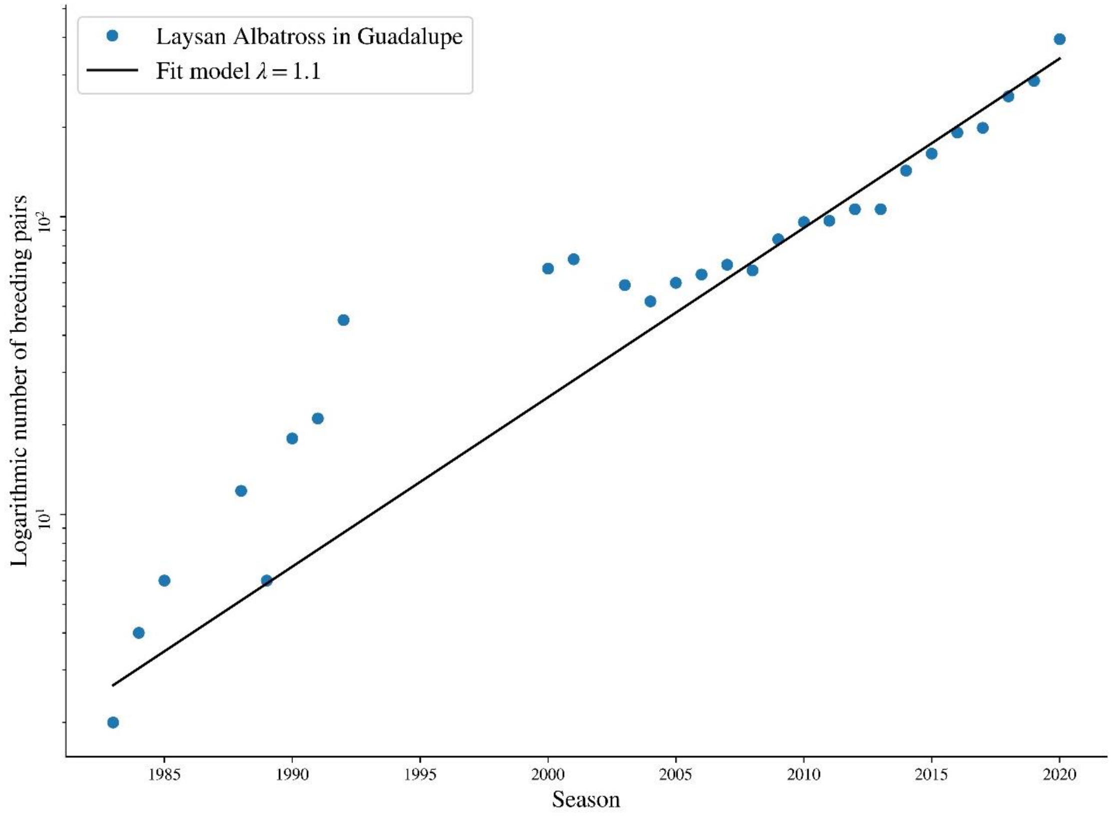

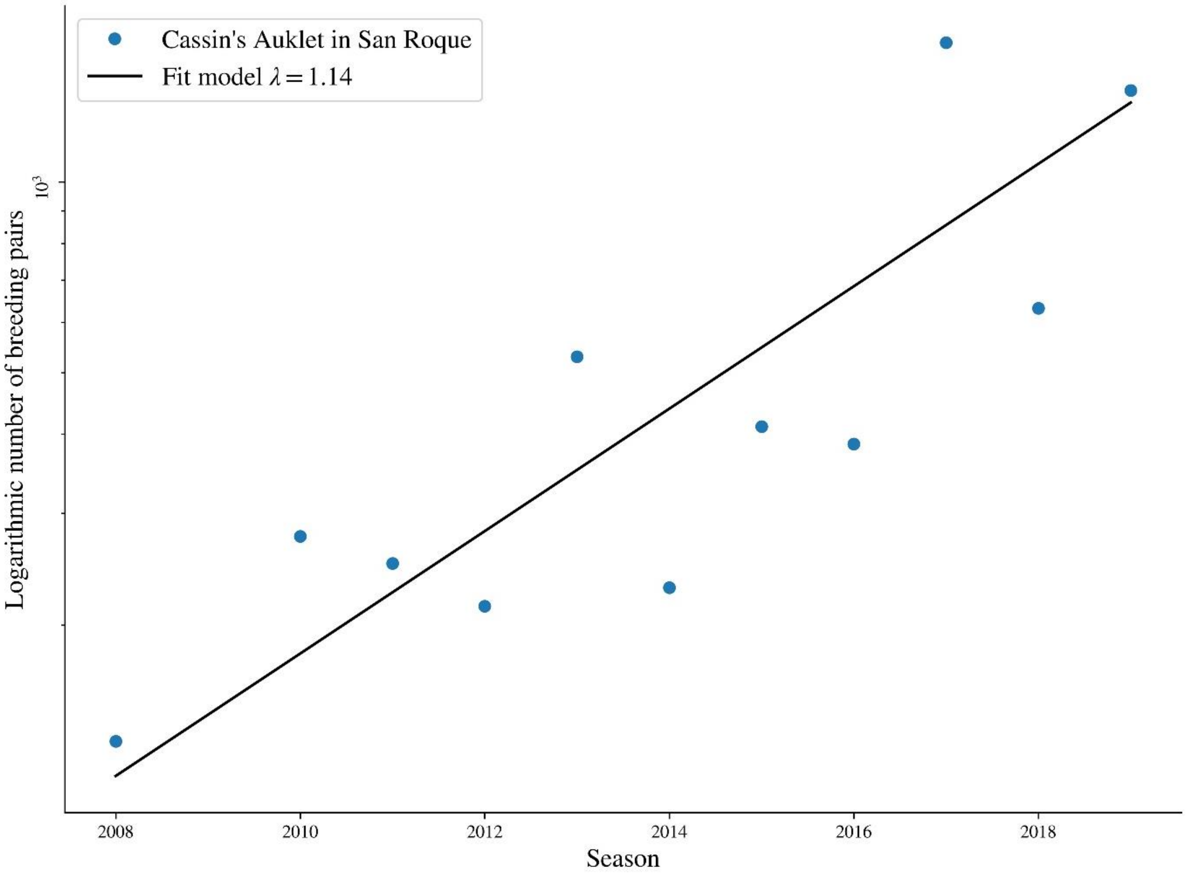

S1 Appendix Fig A. Example plots from our tests showing that the data follow a log-linear trend.

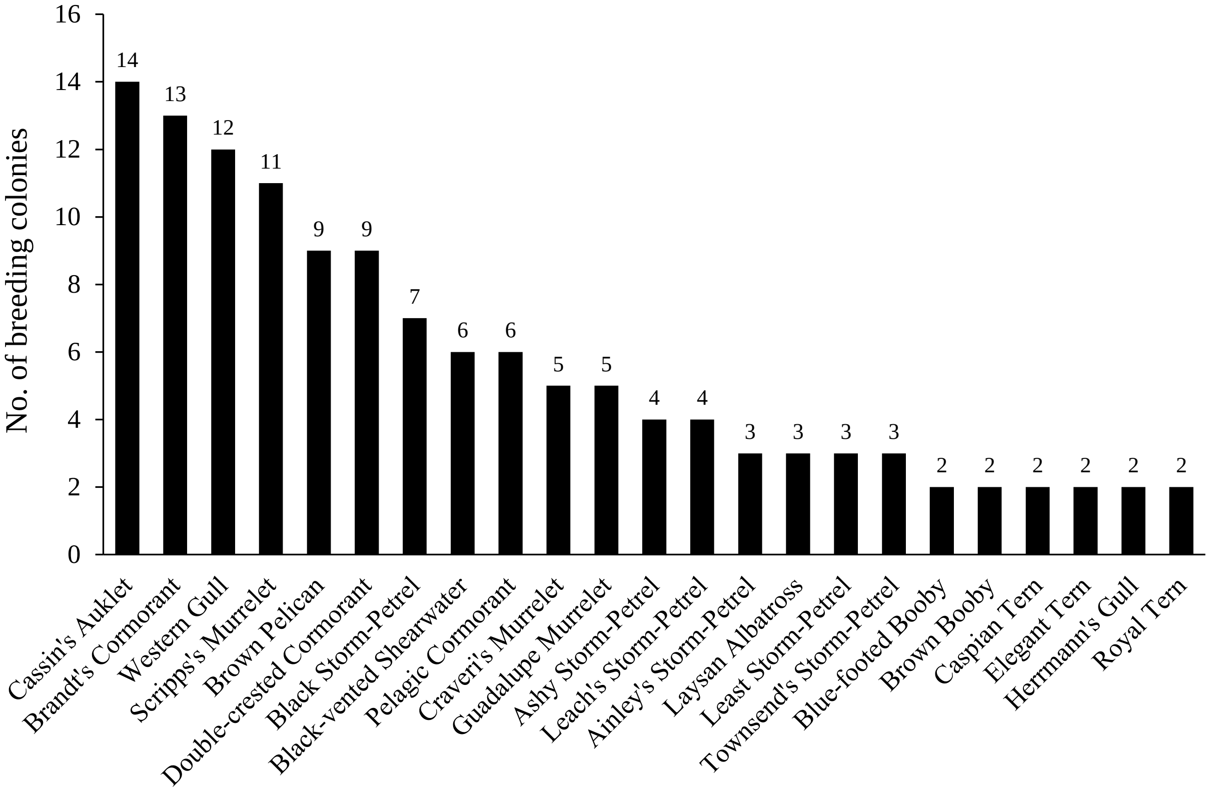

S1 Appendix Fig B. Number of breeding colonies ($N = 129$) for each of the 23 seabird species on the Baja California Pacific Islands.

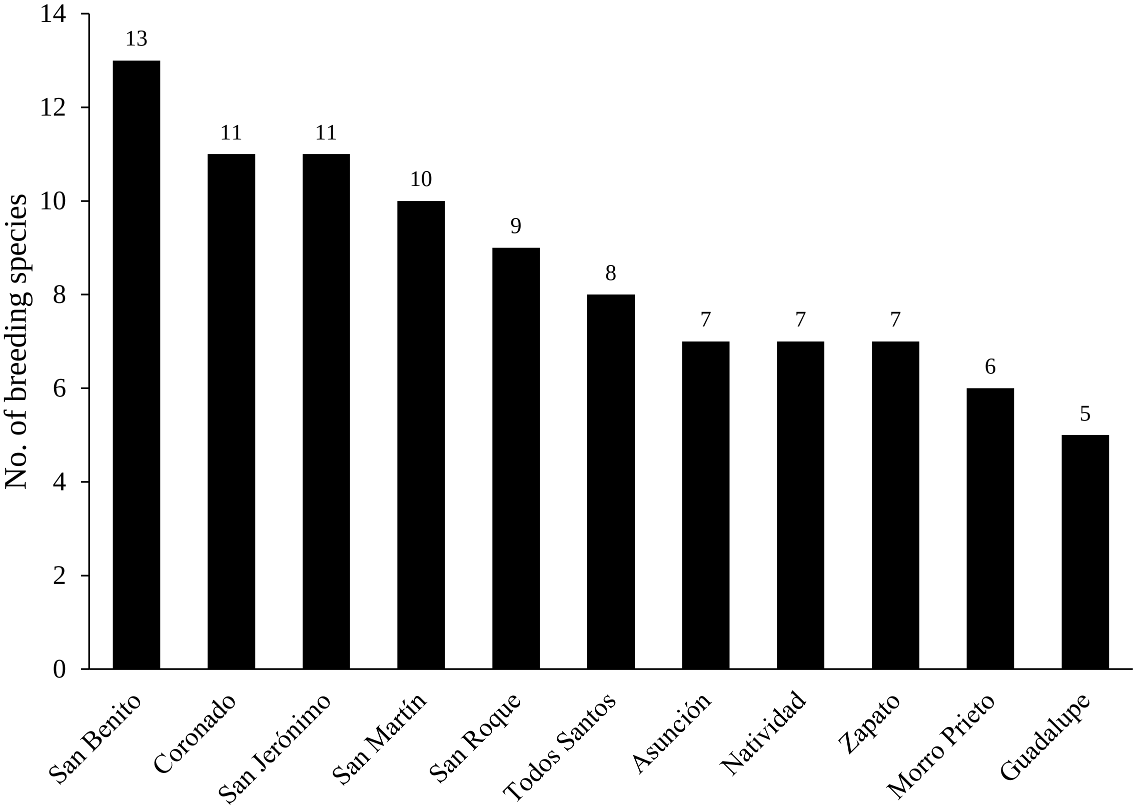

S1 Appendix Fig C. Number of breeding species per island at the Baja California Pacific Islands.

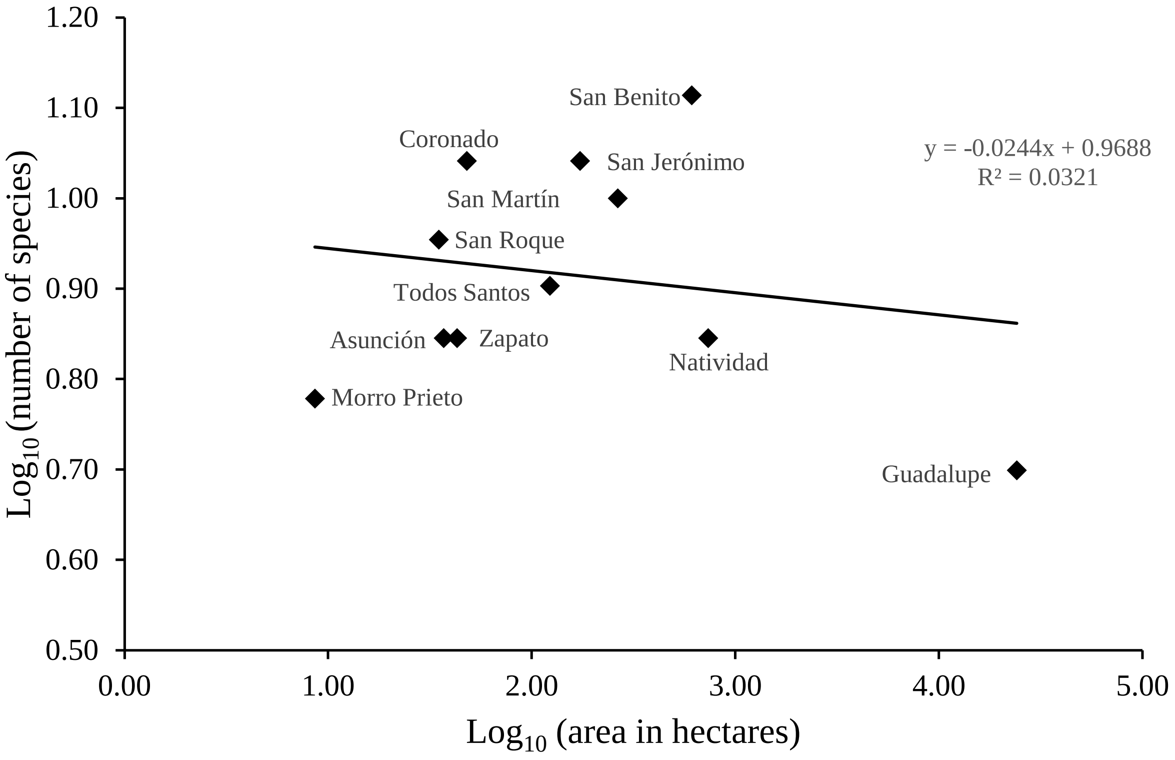

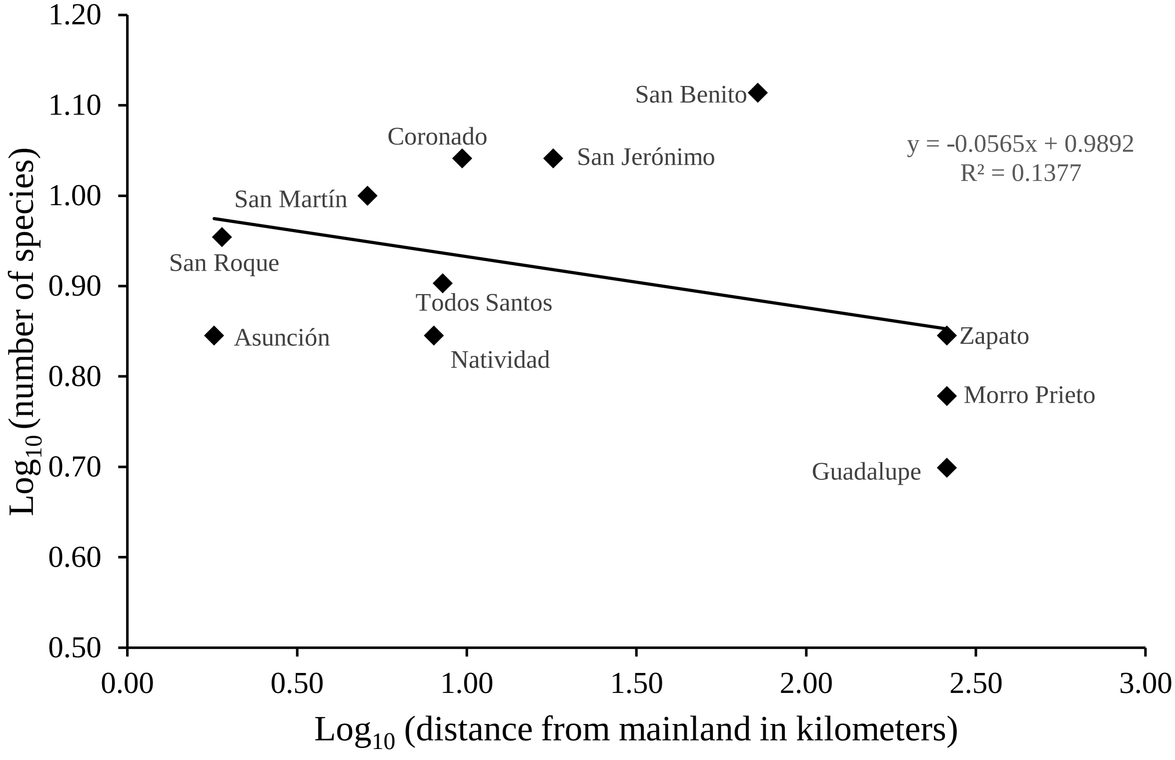

S1 Appendix Fig D. Relationship between seabird species richness and island size (top), and between seabird species richness and distance from the mainland (bottom) for the Baja California Pacific Islands.
